# Valproic Acid, a Histone Deacetylase Inhibitor, in Combination with Paclitaxel for Anaplastic Thyroid Cancer: Results of a Multicenter Randomized Controlled Phase II/III Trial

**DOI:** 10.1155/2016/2930414

**Published:** 2016-09-27

**Authors:** Maria Graziella Catalano, Mariateresa Pugliese, Marco Gallo, Enrico Brignardello, Paola Milla, Fabio Orlandi, Paolo Piero Limone, Emanuela Arvat, Giuseppe Boccuzzi, Alessandro Piovesan

**Affiliations:** ^1^Department of Medical Sciences, University of Turin, Turin, Italy; ^2^Oncological Endocrinology, A.O.U. “Città della Salute e della Scienza di Torino” Hospital, Turin, Italy; ^3^Transition Unit for Childhood Cancer Survivors, A.O.U. “Città della Salute e della Scienza di Torino” Hospital, Turin, Italy; ^4^Department of Drug Science and Technology, University of Turin, Turin, Italy; ^5^Section of Endocrinology, Division of Internal Medicine, Department of Clinical and Biological Sciences, University of Turin, Turin, Italy; ^6^Endocrinology, Diabetes, and Metabolism Unit, A.O. Ordine Mauriziano di Torino, “Umberto I” Hospital, Turin, Italy

## Abstract

Anaplastic thyroid cancer (ATC) has a median survival less than 5 months and, to date, no effective therapy exists. Taxanes have recently been stated as the main drug treatment for ATC, and the histone deacetylase inhibitor valproic acid efficiently potentiates the effects of paclitaxel* in vitro*. Based on these data, this trial assessed the efficacy and safety of the combination of paclitaxel and valproic acid for the treatment of ATC. This was a randomized, controlled phase II/III trial, performed on 25 ATC patients across 5 centers in northwest Italy. The experimental arm received the combination of paclitaxel (80 mg/m^2^/weekly) and valproic acid (1,000 mg/day); the control arm received paclitaxel alone. Overall survival and disease progression, evaluated in terms of progression-free survival, were the primary outcomes. The secondary outcome was the pharmacokinetics of paclitaxel. The coadministration of valproic acid did not influence the pharmacokinetics of paclitaxel. Neither median survival nor median time to progression was statistically different in the two arms. Median survival of operated-on patients was significantly better than that of patients who were not operated on. The present trial demonstrates that the addition of valproic acid to paclitaxel has no effect on overall survival and disease progression of ATC patients. This trial is registered with EudraCT 2008-005221-11.

## 1. Introduction

Anaplastic thyroid cancer (ATC) is one of the most lethal solid tumors and it usually has a rapidly fatal clinical course, its median survival being less than 5 months from diagnosis. Although rare (2% of all thyroid cancers), ATC accounts for 14–50% of total mortality for thyroid cancer, with survival rates of 10–20% at one year and less than 2% at ten years [1]. In the last decade, multimodality treatment (surgery if feasible, followed by radiotherapy in combination with various chemotherapeutic drugs) has been recommended as the optimal approach, but the results of such treatment still remain poor without significant impact on the survival rates and important side effects [2, 3]. ATC is classified as stage IV by the International Union Against Cancer and American Joint Commission on Cancer classifications and the use of systemic chemotherapy is frequently recommended independently of the presence of distant metastases. Taxanes (paclitaxel and docetaxel) are considered the first-line chemotherapeutic agents, for ATC treatment [4], also if the promising results obtained in earlier studies [5] were not unanimously confirmed by subsequent reports [6, 7]. Chemotherapy can be particularly harmful in older patients, like those typically affected by ATC [8], and a dose reduction due to hematological and neurological toxicity is often necessary; therefore its use may be cumbersome in the clinical practice. The use of biological therapy, either with tyrosine kinase inhibitors or with other targeted therapies (e.g., fosbretabulin and everolimus), was advised in ATC but the results of clinical trials have been discouraging [9]. As a consequence, no standard effective medical therapy for ATC exists to date. Deacetylase inhibitors (DCI) represent a class of therapeutic agents with broad activity against cancer cells, namely, cytotoxic effects, induction of cell differentiation, and synergism with established and experimental cancer therapeutics [10]. In preclinical studies, the combination therapy of DCI with chemotherapeutic agents enhanced drug therapeutic efficacy against ATC. Suberoylanilide hydroxamic acid (SAHA), approved by FDA for the treatment of cutaneous T cell lymphoma, has shown anticancer activity* in vitro* and* in vivo* alone or in combination with paclitaxel, doxorubicin, or Paraplatin [11], but unfortunately it resulted as ineffective in a phase II study in patients with advanced thyroid cancer [12]. Belinostat (PXD101) represses thyroid cancer proliferation and exerts synergistic effects in combination with doxorubicin and paclitaxel [13]. In both* in vitro* and* in vivo* models of ATC, panobinostat (LBH589) has shown cytotoxic activity [14], suppressing migration and invasion [15] and inducing radioiodine cytotoxicity [16]. Very recently, SP600125, a small compound acting on the ROCK/HDAC6 pathway, has been demonstrated to selectively induce cell death in undifferentiated thyroid cancer cell lines, making it a good candidate for developing new drugs against ATC [17]. We previously demonstrated that the DCI valproic acid (VPA) efficiently potentiates the effects of doxorubicin [18] and paclitaxel (TAX)* in vitro* [19]. These findings provided the rationale for the present randomized, controlled, phase II/III, multicenter clinical trial with the aim of assessing the efficacy and safety of TAX with (experimental arm) or without VPA (control arm) for the treatment of patients affected by ATC.

## 2. Patients and Methods

### 2.1. Patients

The study was performed across 5 centers in northwest Italy between 2009 and 2012, on patients of both genders affected by ATC and eligible for treatment with TAX. The diagnosis was obtained by cytological examination of specimens following fine-needle aspiration and was confirmed by histologic examination in all patients who underwent surgery. Examination was performed by two different pathologists with proven experience in thyroid cancer. Inclusion criteria were (a) patients with radiologically or clinically confirmed disease progression during or after multimodal therapy (surgery, if performed, first-line systemic chemotherapy with doxorubicin and cisplatin, and external beam radiation (EBR)); (b) patients not eligible for surgery or EBR because of tumor local invasion or severe comorbidities. Patients had to have an Eastern Cooperative Oncology Group (ECOG) Performance Score <3. Liver function had to be adequate as evidenced by serum total bilirubin <2x the upper limit of normal (ULN) (<3x ULN in patients with liver metastases); AST (aspartate aminotransferase)/ALT (alanine aminotransferase) <3x the ULN for the local reference laboratory (<5x the ULN for patients with liver metastases). Exclusion criteria were liver and/or kidney failure; thrombocytopenia and/or thrombocytopathy; treatment with aspirin or antiplatelet agents; treatment with antipsychotic drugs; impossibility of obtaining an informed consent due to impaired consciousness; pregnant or breastfeeding women; fertile women not taking contraceptive pills. The trial was approved by the local ethic committee for each study site. Patients or their legal representatives needed to be able to read, understand, and provide written informed consent to participate in the trial, according to the Declaration of Helsinki.

### 2.2. Study Design

Patients were openly randomized into two arms: the experimental arm received the combination of TAX (80 mg/m^2^) and VPA (1,000 mg/day); the control arm received TAX alone (80 mg/m^2^). TAX was administered weekly intravenously, dose reduction being allowed in case of hematological or gastrointestinal toxicity; VPA was administered daily p.o. in two divided doses of 500 mg. In the absence of disease progression, patients of both groups were treated up to 18 chemotherapy cycles. Primary outcomes were (1) overall survival and (2) disease progression, evaluated in terms of progression-free survival (PFS). The extent of thyroid neoplasia and secondary lesions were assessed at the beginning of treatment by computer tomography (CT) scans of head, neck, chest, and abdomen. Local recurrence or lymph node metastases were evaluated also by high definition neck ultrasonography (US). CT and US were repeated every 6–8 weeks, to assess the response to treatment according to the modified World Health Organization (WHO) criteria: (a) complete response (CR), disappearance of all known lesions and no new lesions; (b) partial response (PR), ≥50% decrease in the sum of the products of diameters of lesions; (c) disease stabilization (SD), absence of CR or PR, without any evidence of disease progression; (d) progressive disease (PD), ≥25% increase in the size of one or more measurable lesions or the appearance of new lesions. Modifications of WHO criteria refer to the persistence of response for 4 weeks after evaluation; this last criterion cannot be applied to ATC, whose clinical doubling time is very rapid, up to three days. Clinical follow-up was performed every two weeks and will last up to 12 months. (3) Quality of life is evaluated using the EORTC QLQC30 v3 ranking every six weeks.

### 2.3. Safety

Hemogram, serum biochemistry, and electrolyte levels were measured before each chemotherapy cycle and when clinically indicated; toxicity was evaluated according to the Common Toxicity Criteria (CTC). A temporary suspension of TAX treatment, followed by a 25% reduction at the restart, was foreseen in the presence of mucositis (Grade 3); hematologic toxicity (Grade 2); gastrointestinal toxicity (Grade 3); neurotoxicity (Grade 3); cardiac toxicity (Grade 3); muscle and skeletal toxicity (Grade 3). In the presence of Grade 4 toxicity or in case of severe allergic reaction, treatment was definitively stopped.

### 2.4. Pharmacokinetics

The secondary outcome of the study was the pharmacokinetics of TAX. During the first chemotherapy cycle, the pharmacokinetic parameters of TAX (*C*_max_, AUC, *K*_el_, *t*_1/2_, clearance, and *V*_ss_) were evaluated in 20 patients (10 receiving TAX; 10 receiving VPA + TAX) by a noncompartmental pharmacokinetic analysis performed by Kinetica 2000 4.1.1 software (InnaPhase Corp., Philadelphia, USA). Blood samples were collected in heparinized tubes, from a large vein in the arm opposite that receiving the drug infusion, at the following times: time 0 (immediately before TAX infusion), 1.5 hours (half infusion), 3 hours (end of infusion), 4 hours, 5 hours, and 6 hours. Blood samples were centrifuged immediately for 10 min at 1,000 ×g at room temperature and the resulting plasma was frozen and stored at −80°C until analysis.

TAX plasma concentrations were measured according to a procedure described elsewhere [20]. Briefly, a volume of 200 *μ*L of acetonitrile, 300 *μ*L of deionized water, and 5 mL of tertbutylmethylether was added to 100 *μ*L of plasma samples in borosilicate glass tubes. The mixture was vortexed for 30 sec and centrifuged at 2,500 ×g for 15 min. Upon centrifugation, 3 mL of the organic layer was transferred to a clean glass tube and evaporated to dryness under nitrogen. The residue was then reconstituted with 200 *μ*L of 60% acetonitrile in deionized water and mixed on a vortex mixer for 90 sec; then 100 *μ*L of the reconstituted sample was injected into the HPLC system equipped with a Symmetry C18 column (250 × 4.6 mm i.d., particle size 5 *μ*m) and a Symmetry C18 guard column supplied by Waters (Vimodrone, Milan, Italy). Chromatographic analysis was performed at room temperature (20 ± 2°C) by isocratic elution with a mobile phase consisting in a mixture of acetonitrile—0.1% phosphoric acid in deionized water (60 : 40, v/v) (Milli-Q Plus System, Millipore, Milford, MA, USA) at a flow-rate of 1.0 mL/min. UV detection wavelength was 227 nm and TAX retention time was about 10 min. TAX concentrations were determined from the peak area ratios versus a standard curve obtained with the same procedure. The limit of quantitation for this method was 5 ng/mL.

### 2.5. Valproic Acid Levels

Serum levels of VPA were evaluated in 11 patients at 2 and 4 weeks after they started VPA treatment. Drug measurement was performed using the Dimension Vista System (Siemens, Healthcare, Erlangen, Germany). The therapeutic range was 50–100 g/mL.

### 2.6. Statistics

Median and range were reported for demographic and clinical variables descriptions. Probabilities of overall survival (OS) and PFS associated with combination treatment (VPA + TAX) versus TAX treatment alone were estimated from the time of enrollment using Kaplan-Meier method; log-rank test compared distributions of the two groups. Assuming that the predicted 1 year overall survival of patients treated with TAX alone is 0.1% and that the addition of VPA may lead to an overall survival of 20%, 22 patients could permit us to demonstrate a significant difference in the primary outcomes, accepting an error of 0.05 and 80% power. The occurrence of death within 365 days from enrollment was considered the event for estimated OS. Patients who were alive after 365 days were censored for the survival analysis. PFS was defined as the time to disease progression as evaluated by CT. Patients with clinical events unrelated to treatments or disease progression were censored.

The Mann–Whitney nonparametric test was used to compare pharmacokinetic values of the two different groups of patients; statistical evaluation was performed using Instat 3.05 software (Graphpad, San Diego, USA).

## 3. Results

### 3.1. Patients

Patient characteristics are reported in Table 1. The trial enrolled 25 patients; 14 patients were randomized to the TAX arm; 11 patients were randomized to the VPA + TAX arm. 13 patients were males (8 in the TAX arm and 5 in the TAX + VPA arm); 12 patients were females (6 in the TAX arm and 6 in the TAX + VPA arm). At diagnosis, median age was 70 years (range 44–83) (73.5 for the TAX arm [range 62–83] and 66 for the TAX + VPA arm [44–80]). Disease stage was IV-A in 2 patients (1 in the TAX arm and 1 in the TAX + VPA arm), IV-B in 6 patients (1 in the TAX arm and 5 in the TAX + VPA arm), and IV-C in 17 patients (12 in the TAX arm and 5 in the TAX + VPA arm). Lymph node metastases were present in 9 patients (5 in the TAX arm and 4 in the TAX + VPA arm); lung metastases were present in 9 patients (7 in the TAX arm and 2 in the TAX + VPA arm). No brain metastases were present at diagnosis. 8 patients (7 at stage 4C and 1 at stage 4A) did not undergo thyroidectomy (6 in the TAX arm and 2 in the TAX + VPA arm); while 17 patients (10 at stage IV-C, 6 at stage IV-B, and 1 at stage IV-A) underwent surgery (8 in the TAX arm and 9 in the TAX + VPA arm). One patient, randomized to the TAX + VPA arm, died only 6 days after the informed consent and, therefore, was excluded from the statistical analysis.

### 3.2. Efficacy

**Figure 1:**
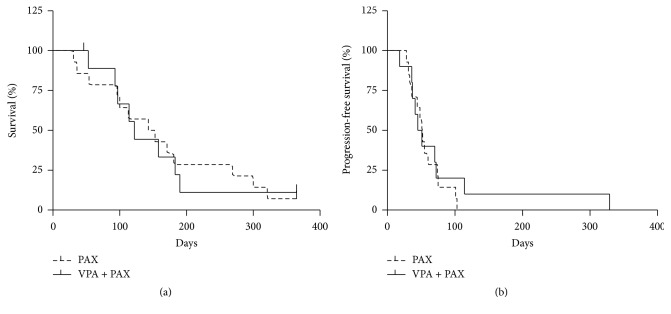
Kaplan-Meier plots of overall survival (a) and progression-free survival (PFS, (b)) of patients with anaplastic thyroid cancer treated with VPA + TAX (-) versus TAX (- -).

**Figure 2:**
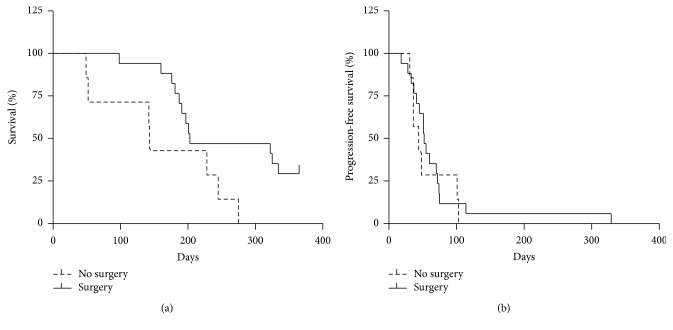
Kaplan-Meier plots of overall survival (a) and progression-free survival (PFS, (b)) of patients with anaplastic thyroid cancer who underwent total thyroidectomy (-) versus no thyroid surgery (- -) (*P* < 0.05).

Median survival of the 14 patients treated with TAX was 148 days, whereas median survival of the 10 patients treated with VPA + TAX was 122 days (*P* = 0.9) (Figure 1(a)). Median time to progression in the TAX group was 51 days, versus 48 days in the VPA + TAX group (*P* = 0.6) (Figure 1(b)). Median survival of patients who had been operated on was significantly better than that observed of patients who had not (203 days versus 143 days, *P* = 0.03) (Figure 2(a)). Median time to progression of patients who had been operated on was not significantly better than that observed in patients who had not (52 days versus 44 days, *P* = 0.6) (Figure 2(a)).

### 3.3. Safety

The oral administration of 1,000 mg valproate given once daily induced the raise of valproate blood levels to values within the proper therapeutic range for epilepsy treatment after two weeks of treatment (67 g/mL; range 50–72). No increase in serum values of liver enzymes was observed.

For 24/25 patients enrolled in the study, the scheduled dose of TAX was maintained. In one patient, TAX was reduced to 75%, as a consequence of allergic reaction during the first TAX infusion. No severe adverse effect was observed. The median duration of treatment was not statistically different between the group treated with VPA + TAX (73 days; range 36–183 days) and the group treated with TAX alone (102 days; range 31–300). For all the patients enrolled, therapy was administered as planned by study design, until clinical progression or death.

### 3.4. Pharmacokinetics

**Figure 3:**
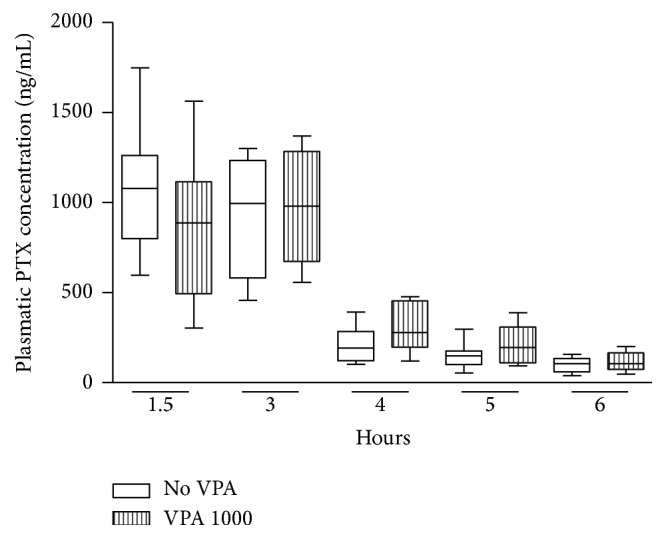
Median plasmatic paclitaxel concentrations for patients treated with TAX alone (VPA 0) or with VPA + TAX (VPA 1000). Boxes = 25th and 75th percentiles; bars = min and max values.

Figure 3 shows the median plasma profile of TAX for patients treated with TAX alone or with VPA + TAX.  Table 2 summarizes the medians of the main pharmacokinetic parameters of TAX obtained from the plasmatic curves for both control and experimental groups. The results of Mann–Whitney test showed no difference between the two groups, demonstrating that the coadministration of VPA did not influence the pharmacokinetics of TAX.

## 4. Discussion

An effective treatment for ATC, one of the most lethal cancers, has not been established yet [21, 22]. The American Thyroid Association guidelines [23] recommend a multimodal approach including surgery, radiation, and systemic chemotherapy for patients with stage IV-A/IV-B ATC, and this approach showed some efficacy also in patients with stage IV-C disease in our experience [24]. More recent NCCN guidelines recommend docetaxel/doxorubicin schemes with or without EBR therapy and suggest TAX 60–90 i.v. mg/m^2^/weekly as the most effective single therapeutic agent. Both guidelines conclude that current systemic treatments lead to poor outcome and fail in improving survival or PFS. Several novel therapies have been evaluated in clinical trials, such as vascular disrupting agents (fosbretabulin, combretastatin, and crolibulin) and multitargeted therapies (bevacizumab, a number of tyrosine kinase inhibitors such as sorafenib, sunitinib, imatinib, and pazopanib). None of these agents showed any relevant effect on survival or PFS, being similar to those obtained with the traditional multimodal approach. These discouraging results were obtained mainly from observational studies performed in small series of patients. The present study is a rare example of an interventional randomized trial comparing one established treatment (TAX) with a combination one (TAX plus VPA).

The decision to combine VPA with TAX in the present clinical trial stems from data obtained (by our group) with VPA as histone deacetylase inhibitor. The advantage of using VPA, a potent anticonvulsant widely used to treat epilepsy and mood disorders that acts as DCI at therapeutic concentrations [25, 26], is that it produces only mild adverse effects in humans, even when serum levels exceed the normal therapeutic range. Besides histones and other nonhistone proteins, *α*-tubulin is also a substrate of histone deacetylases [27]. In line with this, we reported elsewhere that the combination of VPA with TAX enhanced TAX cytostatic and cytotoxic activity in ATC cell lines [19]. The effect was reached with VPA at a dose of 0.7 mM, which corresponds to plasma levels within the therapeutic range in patients treated for epilepsy and is without any serious side effects. Suppression of microtubule dynamics is recognized as the mechanism by which TAX blocks mitosis and kills tumor cells [28]: rapid dynamics ensure timely and accurate chromosome movement; and our previous* in vitro* data clearly showed that VPA induced tubulin acetylation and maximally potentiated the acetylating activity of TAX. Beyond our previous* in vitro* data, several groups have investigated the possibility of combining VPA with different chemotherapeutic agents in patients with leukaemia and solid tumors. VPA has been shown to potentiate the activity of doxorubicin in patients with breast cancer [29]; moreover, in patients with metastatic tumors, the combination of VPA and epirubicin has been shown to be effective and well tolerated [30]. Finally, data are available from phase I/II clinical trials with VPA and 5-azacitidine in myelodysplastic syndrome [31]. On the contrary, to the best of our knowledge, no other clinical trials are available on the efficacy of the combination of VPA and TAX.

Unfortunately, the encouraging results obtained* in vitro* on the enhancing action of VPA on TAX were not confirmed in the clinical setting of our ATC patients. In fact, mean overall survival and PFS were comparable in the 2 groups of patients, and they did not differ from those reported in other previous series of patients treated with traditional multimodal treatment. This observation is not new, since, for several therapeutic agents such as fosbretabulin [32, 33] and combretastatin [34] and, more recently, for sorafenib [9] and pazopanib [35], the promising effects described in experimental models of ATC were not confirmed by strong outcomes in clinical trials. The median survival observed in our study did not differ from that reported in clinical trials of fosbretabulin plus paclitaxel/carboplatin (4.7 months), sorafenib (3.9 months), combretastatin (5.2 months), and pazopanib (111 days). The number of patients treated in a second-line setting was greater in the VPA + TAX arm (8/10) than in the TAX arm (4/14) but the performance status did not differ between the two groups. However, the possibility that more chance of resistance to cytotoxic agents in patients treated in second-line setting could explain the lack of significant differences between VPA + TAX and TAX arm may not be inferred by our data and remain speculative.

Patients treated with TAX plus VPA did not experience more side effects than those treated only with TAX. Drug interaction between TAX and antiepileptic drugs has been previously described [36]. TAX is metabolized extensively by CYP2C8 and, to a smaller extent, by CYP3A4; furthermore TAX is a substrate of P-gp. TAX is therefore susceptible to interactions with drugs that induce or inhibit these proteins. As some preclinical* in vitro* and* in vivo* experiments demonstrated that VPA is able to slightly inhibit CYP3A4 (Ki = 7975 *μ*M) and to induce the overexpression of P-gp, therefore a pharmacokinetic interaction between the two drugs could occur in the patients of the experimental arm [37, 38]. Our results demonstrated that pharmacokinetic parameters of TAX showed no difference between the control and experimental arms, suggesting no pharmacokinetic interaction of VPA that could influence TAX clinical efficacy.

In conclusion, regardless the promising preclinical results, the present trial demonstrated that the combination of VPA and TAX was not superior to TAX alone. Nevertheless, our results did not differ from those observed in other studies in patients with advanced ATC.

## Figures and Tables

**Table 1:** Patient characteristics.

	TAX	VPA + TAX
Number of enrolled patients	14	11

*Gender*		
(i) Male	8	5
(ii) Female	6	6

*Age (years)*		
(i) Median	73.5	66
(ii) Range	62–83	44–80

*Disease stage*		
(i) 4A	1	1
(ii) 4B	1	5
(iii) 4C	12	5

*Distant metastasis *		
(i) Lymph nodes	5	4
(ii) Lung	7	2

*Previous therapy*		
(i) Radiotherapy	6	7
(ii) Chemotherapy	4	7
(iii) Thyroidectomy (incomplete)	7 ^*∗*^R0/R1 (1 R2)	8 ^*∗*^R0/R1 (1 R2)

^*∗*^R0/R1: macroscopically complete resection; R2 resection with minimal macroscopical residual tumor.

**Table 2:** Pharmacokinetic parameters.

Pharmacokinetic parameters	Median (range)		*P* value (Mann–Whitney)
	TAX	VPA + TAX	
*C*_max_ (ng/mL)	1155 (767–1749)	1134 (608–1565)	0.85
AUC_tot_ (ng × h/mL)	3490 (2177–4321)	3434 (2050–4137)	0.68
*K*_el_ (1/h)	0.34 (0.18–0.56)	0.49 (0.41–0.56)	0.05
*t*_1/2_ (h)	2.03 (1.24–3.94)	1.42 (1.25–1.69)	0.05
Cl (mL/h)	39858 (30189–71196)	41819 (30898–71012)	0.91
*V*_ss_ (mL)	52138 (43138–147388)	61452 (34164–142622)	0.58
